# The Use of Video Conferencing for Persons with Chronic Conditions: A Systematic Review

**DOI:** 10.4236/etsn.2016.52005

**Published:** 2016-06

**Authors:** Jennifer A. Mallow, Trisha Petitte, Georgia Narsavage, Emily Barnes, Elliott Theeke, Brian K. Mallow, Laurie A. Theeke

**Affiliations:** 1 School of Nursing, West Virginia University, Morgantown, WV, USA; 2 Sovern Run, LLC, Albright, WV, USA

**Keywords:** Video Conferencing, Chronic Conditions, Outcomes of Care

## Abstract

The purpose of this paper is to present a systematic review of studies that used Video Conferencing (VC) intervention for common chronic conditions. Chronic conditions account for the majority of poor health, disability, and death, and for a major portion of health-care expenditures in the United States. Innovative methods and interventions are needed to enhance care and management, improve access to care, improve patient outcomes, narrow health disparities and reduce health-care costs. Video Conferencing could be particularly relevant in improving health, care management, access and cost in the care of chronic illnesses. A comprehensive literature search process guided by the PRISMA statement led to the inclusion of 27 articles measuring video conferencing, at least one chronic illness, and patient outcomes for adults living in a community setting. While VC has been found to be feasible and effective, a low number of randomized controlled trials limit evidence. In addition, studies in this review were not designed to address the question of whether access to care in rural areas is improved through VC. Hence, more research is needed.

## 1. Introduction

Living with multiple chronic conditions (MCC) compounded by experiencing health disparity is extremely challenging. A chronic condition is one that persists a year or longer and requires ongoing medication, attention, and/or limits activities of daily living [1]. Health disparity is an individual’s ability to achieve good health outcomes, which can be affected by ethnicity, gender, age, disability, socioeconomic status, and geographic location, particularly for those living in rural areas [2] [3]. Chronic conditions account for the majority of poor health, disability, and death, and for a major portion of health-care expenditures in the United States (US) [4]. Approximately half (117 million) of US adults have at least one chronic condition, and 1 in 4 adults have multiple chronic conditions [5]. Individuals with MCC may have difficulty achieving goals of treatment due to the complexity of treatments, conflicting advice for each condition, and co-existing health disparities [6]-[9]. Therefore, persons with MCC and health disparities living in rural areas are at higher risk for limited access to care which can result in poor chronic condition control and decreased self-management [10] [11].

Hence, treating MCC while also addressing health disparity is a complicated issue for healthcare providers. This complexity has been defined as the gap between the individual’s needs and the capacity of healthcare services to support those needs [6]. Innovative methods and interventions are essential to enhance the care and management of MCC, improve access to care, improve patient outcomes, narrow health disparities and reduce healthcare costs [12]. Use of technology to treat MCC has been suggested [7] because technology can improve the capability to monitor patients, coordinate care services for patients seeking multiple healthcare providers in different geographic locations, and manage patient symptoms [13]. Video Conferencing (VC) offers a potential solution to this complexity by incorporating audio/visual enhancements to technology-based interventions for the treatment of individuals with health disparity and MCC.

Video conferencing is a communication technology that allows two or more locations to connect in real-time two-way video and audio transmission [14]. This type of technology enables patients to access healthcare services, by using the internet, without the burden of distant travel. Eliminating health disparities such as advanced age, physical disability, and isolated geographic location are possible through this technology. Video Conferencing could be particularly relevant in the context of MCC care when there are high levels of unmet need, and demands on services are likely to increase [5]. The purpose of this paper is to present a systematic review of studies that used Video Conferencing intervention for common chronic conditions.

## 2. Methods

The PRISMA method [15] guided the development of this systematic review. A comprehensive literature search was conducted using EBSCOHost with the following databases selected: Academic Search Complete, CINAHL with Full Text, ERIC, MEDLINE, PsycARTICLES, and PsycINFO. The search term “Video Conferencing” was used and limits were set to include only peer-reviewed studies of adults written in the English Language. The literature search began on 5/1/2015 and concluded on 7/16/2015. The search netted 460 articles; 162 duplicates were eliminated and 298 abstracts were reviewed with the following inclusion criteria: 1) use of Video Conferencing discussed in the abstract, 2) included patients, 3) adults, 4) at least one chronic illness discussed in the abstract, and 5) outpatient/community setting. Articles were excluded if the abstract did not meet these criteria. Three authors completed the literature search (JM, BKM, & ET) to ensure accuracy of the search. Forty abstracts met the inclusion criteria and these full text articles were obtained. The Rosswurm literature critique worksheet [16] was used to review each article in order to reduce the risk of bias of individual studies at the study level and also across all identified studies. The full text article and the literature critique worksheet were reviewed for accuracy by all authors. The results of each critique were then placed into a literature matrix, which was again reviewed by each author for accuracy. The matrix includes the authors, year of publication, location of the study, aim of the study, study design, type of video conferencing, sample size, data collection methods, chronic disease focus, and results of the study.

After reading and critiquing full articles, 13 articles did not meet the inclusion criteria and were eliminated from this review. One article did not fit the definition of VC used in this article [17]. Two articles explained the potential of VC but did not report patient outcomes [18] [19]. Four articles were study protocols and did not report data [20]-[23], three articles were commentaries or letter’s to the editor [24] [25], and four articles measured provider but not patient outcomes [26]-[29]. Finally, the Cochrane Library was searched for a review on video conferencing, but no applicable results were found.

## 3. Results

**Figure 1:**
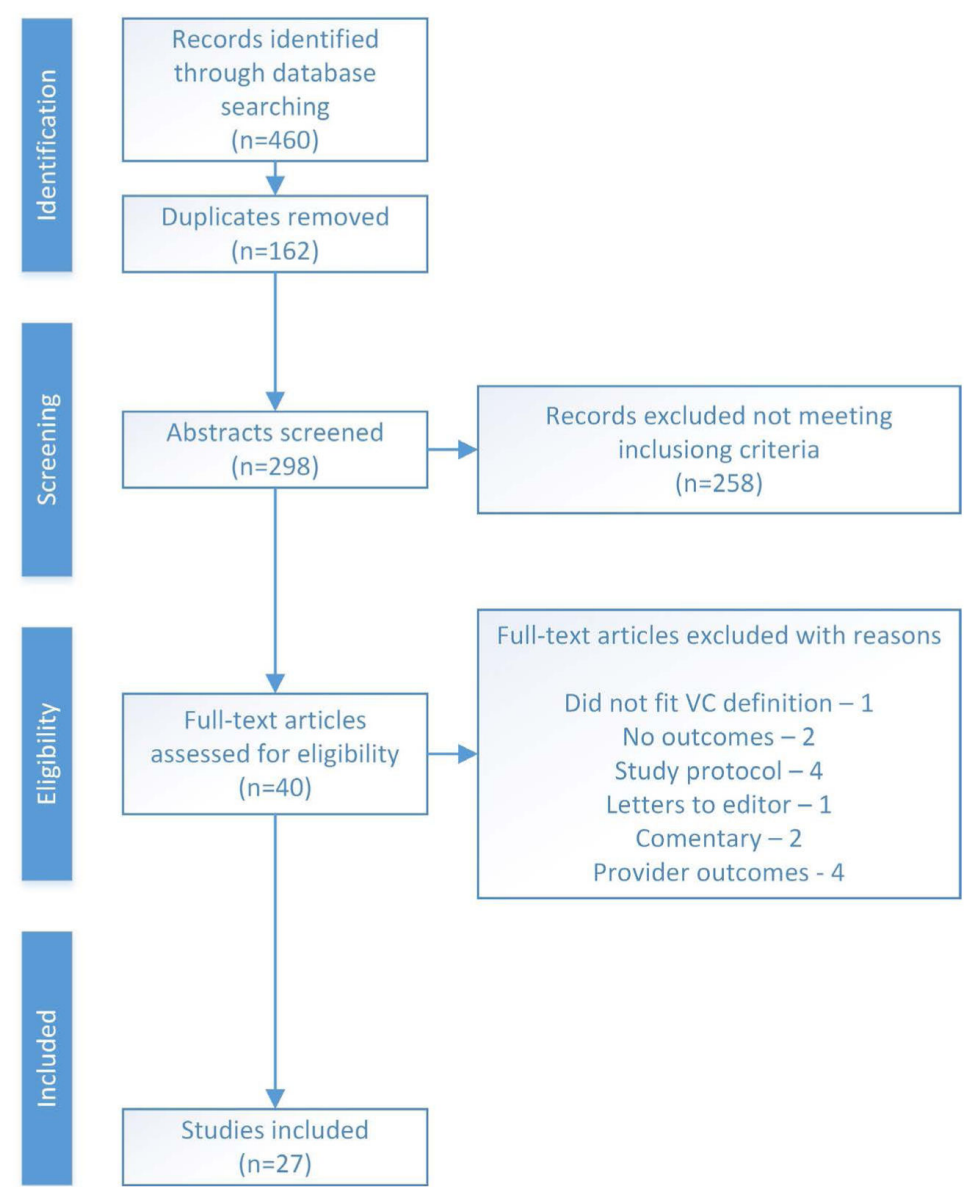
Literature search [insert here from uploaded. jpg file].

A total of 27 articles are included in this review (see Figure 1). The majority of studies took place in The United States (n = 11) and Canada (n = 9). While, a few articles were set in Australia (n = 3), The United Kingdom (n = 3), and China (n = 1). Limits were not set for year published while searching the literature. However, all studies in this review were published within the last 15 years and the majority were published in the last 5 years (n = 16). Of the reviewed articles, three were review articles and eight were randomized controlled trials. The remaining articles were feasibility or quasi-experimental studies (n = 10), descriptive observational studies (n = 3), one cost analysis, one purely qualitative article and one retrospective chart review. The sample size for articles ranged from 2 – 249 participants with the majority of studies having 50 or less participants (n = 14). The type of connection used for the video conferencing was often web based (n = 14) especially in the last 5 years (see Table 1).

### 3.1. Results Overview

Videoconferencing has been used in many different areas of healthcare and has been generally successful in improving patient outcomes. The types of chronic illness that researchers evaluated using VC included: mental health issues (n = 11), neurological (n = 6), long term care patients (n = 2), oncology (n = 2), rehabilitation (n = 2), obesity (n = 1), angina/heart failure (n = 1), diabetes (n = 1), Human Immunodeficiency Virus (n = 1). Video Conferencing was shown to be feasible, result in positive patient experiences, improve outcomes, decrease hospital days, be similar to in-person care, decrease cost, and improve caregiver quality of life [23] [25] [30]-[52]. Two studies found VC to be less efficient or potentially not feasible in certain populations (n = 2) [53] [54].

The studies that found VC to be ineffective attributed the lack of efficacy to the age of the participants, technical difficulty, or a desire to have in-person sessions. A study of real-time web-based monitoring on two patients recovering from strokes was done to help improve their walking ability. One individual strongly favored the technology and the other strongly opposed it due to wanting the primary therapist in person at all times [53]. A study on in-home video conferencing for geriatric rehabilitation was not successful for a variety of reasons including vision and hearing impairment, client anxiety and stress, dementia, cluttered home environment and in-home assistance being needed to operate the equipment and the need to remain with the patient due to high risk for falls [54]. The authors of this study recommended use of video conferencing in a geriatric population that is higher functioning with fewer co-morbidities. All of these studies had limitations of very small sample sizes. Video conferencing should be tested with larger sample sizes to effectively determine its utility.

### 3.2. Mental Health

Most of the literature on use of VC is related to mental health and neurological disorders. Ten different studies report the use of VC in the care of patients and families with dementia, neurodegenerative disorders, and mental health issues [33]-[35] [38] [41] [43]-[45] [48] [52]. Most of these studies had positive results, including being more cost effective and time efficient for both the healthcare provider and patient [34] [35]. However, one study had mixed results. Chua (2001) compared in-person neurological referrals to VC neurological referrals. The VC group had an increased number of assessments, there were no differences in the number of medications prescribed and patients were satisfied with both types of consultation. However, some participants reported embarrassment related to being on camera [33].

### 3.3. Mental Health Diagnosis & Assessment

Diagnosis and assessment related to mental health and neurological disorders is possible using VC. Amarendran *et al*. conducted a study on the ability to complete an assessment of involuntary movement using VC compared to an in-person assessment for 50 participants who had been exposed to antipsychotic medications for a minimum of 10 years [30]. The measure used was the Abnormal Involuntary Movement Scale (AIMS) and the assessment was conducted with two raters in the room and two other raters watching from a nearby remote location; the raters were rotated for each session. An analysis was conducted using multilevel modeling to assess the extent to which total variance in rating was due to the differences between raters rating the same patient and differences between patient to patient. The mean Global Rating for the in-person assessment was 1.35 (sd = 1.03) and the mean Global Rating for the VC assessment was 1.37 (sd = 0.92). The difference was not significant.

Martin-Khan (2012) evaluated the use of VC for establishing the diagnosis of dementia and reported that in 205 adults (aged 50 or older), the clinical assessment for the diagnosis of dementia using valid and reliable instruments can be accomplished using VC. During intellectual assessments, results show there is no statistically significant difference in test results compared to in-person care [48]. Another study [41] evaluated VC compared to three other interventions: computer assisted treatment, in-person treatment, and no treatment for 103 patients with Acquired Brain Injury (ABI). While the in-person administered training showed better improvements in self-efficacy and in problem-solving, the diagnosis of dementia using VC was feasible, effective and there were no differences in other measures. Thus, a combination of in-person and VC training for patients with ABI may be beneficial.

Another study found that VC and in-person assessment did differ between groups. Temple et al. conducted a descriptive observational study on the use of video conferencing (VC) compared to in-person assessments of persons with intellectual disabilities (ID) [48]. There were 19 participants with a mean age of 38.5 and some form of ID along with no visual or hearing impairment. The participants were assessed using two measures, the Wechsler Abbreviated Scale of Intelligence (WASI) and the Beery-Buktenice Developmental test of visual-motor integration-IV (VMI). Participants had two testing sessions, one over VC and one in-person with a 5 to 21 month time in between with the mean time being 10.4 months. The results from the WASI indicated the visual IQ (VIQ) scores were higher in-person (in-person mean = 70, VC mean = 67). Though the performance IQ (PIQ) scores were higher for the VC group (VC mean = 68.4, in-person mean = 66.3). The full scale IQ (FSIQ) and VMI on average differed less than 1 point (FSIQ diff = −0.8, VMI diff = −0.6). Analyses show that the WASI scores for FSIQ and PIQ had no significant change. The change in the VIQ however was found to be significant and the VMI scores did not differ significantly. The results indicate that using VC to conduct intellectual assessments is effective; however the sub-scoring of the WASI, specifically the VIQ, suggests that this should be done cautiously. Informal questioning revealed that patients and providers both see VC as an acceptable method of performing assessments. However, this study was also limited by its small sample size.

### 3.4. Mental Health Treatment

Treatment of mental health and neurological disorders for both patients and caregivers can be delivered through or assisted by VC. A mixed methods study was conducted with 18 individuals diagnosed with mood and or anxiety disorder using a VC provided cognitive behavioral therapy (CBT) intervention program compared to in-person CBT therapy. The study showed support for use of VC in the provision of CBT compared to in-person CBT as there were no significant differences in the VC group compared to the in-person group [38].

Marzali & Donahue (2006) compared the effectiveness of a VC support group to a no treatment usual care group in a sample of 66 caregivers of older adults with Alzheimer’s disease. The study concluded that the care-givers rated the computer use as a positive experience and that the VC group improved on caregiver stress while the no treatment group reported increased stress. Subsequently, Marzali & Garcia (2011) compared the reported stress and health status of caregivers of persons with dementia using two groups: an internet chat support group that included a caregiver handbook and six educational videos versus internet-based videoconferencing delivered by a clinician that also included the caregiver handbook. In this study of 91 caregivers, both groups improved on self-efficacy and neither group changed in their use of health and social services. However, the VC group had greater improvement in mental health and stress scores.

Grady *et al*. conducted a retrospective chart review to evaluate the effect of telehealth care for remote military populations suffering from mental health conditions [36]. The records reviewed came from two locations, the National Navel Medical Center and the Patuxent River Clinic. These locations were chosen because they were similarly staffed and provided similar access to medical services. The National Navel Medical Center has offered telehealth care for a few years and the Patuxent Clinic did not offer telehealth services, making the Patuxent Clinic an appropriate control. The principal measure being used was the Global Assessment of Functioning and the treatments measured were laboratory studies, number of medications used, adherence, mental status examinations, general number and type of diagnosis. Of the records available, 81 had two or more visits as required to evaluate a change in Global Assessment of Functioning. Thirty of the participants had in-person encounters and 51 had VC encounters. The in-person groups initial mean score was 56 and the final in-person score was 65 with a mean change being 8.4. The VC group’s mean initial score was 54 and the final was 69, with a mean change in being 15.3. The change between VC and in-person groups was found to be significant. The VC group also had significantly more patients who demonstrated adherence (used medications and came to consultation appointments) than the in-person group (VC = 94%, in-person = 89%). It was also determined that the time between appointments was significantly shorter in the VC group with 73% of the appointments within 30 days as compared to 62% within the same time frame in the in-person group.

Grady, 2002, conducted a comparative cost analysis between the currently available methods of providing treatment of mental health issues for military personnel and veterans [35]. The four options examined were using the locally available heath care providers, travel to a military medical center, travel to a local medical facility where there is a circuit riding provider, and using VC to see a remotely located heath care provider. The cost of each service was calculated taking into account the cost to the patient, cost to the military, and cost to the clinic/provider. These costs included travel, missed work/lost time, equipment cost, reimbursement, and operational cost. The total monthly cost calculated revealed that the most expensive was travel to a military medical facility ($6986.72), followed by using the local network of health care providers ($5510.39), then traveling to a local facility where a circuit provider is located ($5421.67) then using VC systems ($4599.73). The primary reason for the reduced cost of telehealth is the estimated reduction in hospitalization costs ($648.50, 14%) along with reduced appointment times, 5 – 10 min, and the removal of much of the travel time for both the patient and provider. The initial installation of a VC system is estimated at $719.71 per month but the reduction of other costs covers the difference.

More recently, Woolf *et al*., (2015) assessed the feasibility of comparing therapies for word finding for persons with aphasia with four groups: remote therapy from University site, remote therapy from clinical site, in-person therapy, and attention control group. Woolf enrolled 21 persons with post-stroke aphasia and reported that participants gave positive ratings for the method of remote delivery. Participants in all therapy groups improved when compared to the attention-control groups but those who received remote therapy from the clinical site were most improved.

A descriptive feasibility study conducted by Azad *et al*., evaluated the use of VC for dementia management in rural areas. The study involved 99 patients who used VC from the Tri-County Mental Health site in Cornwall to see 32 health care providers at the Ottawa Hospital between Nov. 2006 and Nov. 2010. The patients were identified by a geriatrician as being medically stable with noncomplex conditions and presenting with mild cognitive impairment or mild dementia. Only 50 of the 99 patients (51%) provided complete information on these measures. Most (92%) of the patients felt they were able to communicate all desired information to their provider as they could during an in-person visit. The Majority (90%) of patients and providers state they would be willing to use VC again. Of the 32 providers involved, 30 gave feedback and felt many of their appointments would have been canceled had the patient had to travel to the clinic, and 96% felt the VC appointment gave them needed advise related to clinical decision making. Without technical difficulties, which 7 out of the 30 providers experienced, the VC system allowed them to monitor their patients more closely and with greater efficiency.

### 3.5. Lifestyle Modification

Some success has been reported with VC for group and lifestyle intervention programs aimed at preventing diabetes [49], improving obesity [32], and healthy relationship habits [42]. The diabetes prevention study had an on-site program (n = 13) and a VC program (n = 14) that were both 16-weeks in which people with risk factors for diabetes were asked to keep track of fat and calorie intake and record minutes of physical activity. In both groups over 45% of participants achieved the 7% weight loss goal although there was no significant difference between the groups. The implications from this study are that increased participation from multiple sites in a diabetes prevention program is possible. Obesity interventions, such as virtual group visits using VC may assist in overcoming barriers including access to care and yield positive clinical outcomes, such as a decrease in body mass index. Results from a VC study of relationships in women with HIV showed that participants (n = 4) liked the increased and equitable attention among group members as well as reportedly high levels of unity and togetherness in the group. All the women stated that they would prefer VC for the Healthy Relationship program as opposed to an in-person program.

### 3.6. Oncology

Oncology patients have also experienced benefits with use of VC. Tamara and colleagues (2015) conducted a pilot study of a mobile health Pain Coping Skills Training (PCST) protocol for 25 patients with persistent pain from breast, lung, colorectal or prostate cancer who live an average of 69 miles from the medical center. Participants received a tablet computer to access four pre-planned video-conferencing sessions. Thirty to 45 minute video conferencing sessions using skype from the therapists’ office taught patients theoretical content on pain management and coached them through skills-based training including relaxation techniques. Of the 84% (N = 19) of patients who completed the study, all found it feasible and were satisfied with the program quality; 95% said it increased their understanding and over 90% reported pain management skill development. Significant reductions in pain severity, physical symptoms, psychological distress, and pain catastrophizing were found post-intervention.

Kitamura, Zurawel-Balaura & Wong (2010) conducted a systematic review of the literature to evaluate the feasibility of assessing, monitoring, and managing oncology patients via VC. They identified 19 published articles of 15 clinical oncology patient groups: one small randomized controlled trial (RCT); 7 non-randomized with control groups, and 7 case studies, with a total of 709 VC study patients and 346 control patients. Inclusion criteria were that VC included two-way communication for monitoring and was used by physicians or nurses with cancer patients in real time with at least one reported clinical outcome. Reported outcomes included patient satisfaction (although no validated scales were included) and preference for VC consultation, costs, provider satisfaction and convenience. The study supported the feasibility of VC with effectiveness for assessing, monitoring, and managing care of oncology patients. Clinical outcomes were not compromised and time and cost were comparable or reduced.

### 3.7. Follow-Up Care

Video conferencing has also been used in older populations for follow-up care for common chronic conditions and acute exacerbations of chronic conditions. Woodend and colleagues (2008) conducted a RCT to evaluate the effect on healthcare resource use, morbidity, and quality of life, of a 3 month intervention that included VC enhanced telemonitoring for patients with heart failure (HF) or angina. The weekly VC intervention involved a nurse contact in addition to daily telephone transmission of weight, blood pressure and periodic electrocardiograms (EKG) and a 1-year end-of study assessment. Video conferencing in combination with home tele-monitoring was easy to use and had high satisfaction. There were no significant differences in physician visits between the VC and usual care groups. Primary outcome data documented 51% reduced number of hospital readmissions and 61% reduced days in hospital for patients with angina over 3 months and one year (reduced 45%/21%). Video conferencing was also an effective model of care for residents of long-term care facilities to enhance medical decision making for unscheduled conferencing with on call-physicians [23] and to facilitate consultation with specialty care [50].

## 4. Discussion

Although VC has been studied for the past 15 years, evidence of effectiveness is limited by a low number of RCTs with large numbers of participants. Small samples and methodological weaknesses of the studies are major limiters to generalizability of the findings. However, studies of VC have shown feasibility, acceptability, efficacy, and cost effectiveness. In addition, initial assessment of movement and mental health disorders using VC may be different from in person care. However, diagnosis and treatment ability have been found to be the same as in-person care. More research is needed with larger samples and randomization.

The outcomes of this systematic review are that studies are not adequately designed to address the question of whether “access” in rural areas is improved through VC because multiple factors could have influenced the results of the studies. In addition, technical difficulties from what may be outdated technology have limited evidence of mostly moderate quality. Perhaps qualitative research can be used to identify key factors needed to improve access and what components can be done using VC. This could provide a foundation for quantitative studies of VC looking at health care re-admission, co-morbidity, deaths, and cost-effectiveness.

Over the past 5 years, most users of VC have moved to secure web-based systems and away from slower ISDN connections. There is a gap in publication of VC studies in countries outside the United States. Twenty of the 27 studies are conducted in North America where access to faster internet connection and technology is more prevalent. Countries and rural areas that have significant disparity in access to technology and fast internet may find this shift problematic.

Publication bias is a potential issue with this review. Much of the video conferencing technology is being developed by private industry and being used by clinical practices. Both of these groups may not have an academic purpose. Hence, much of the evidence of acceptability, feasibility and efficacy of video conferencing may not be published. This systematic review also contains clinical and methodological heterogeneity across studies. The types of participants, intervention and outcomes differ in each study and variability in study design occurs across studies. Statistical heterogeneity cannot be assessed in this article because a meta-analysis was not completed. Our objective was to complete a comprehensive review of the literature related to video conferencing so that we may conduct a practice change. The underlying systematic review of this literature does not yet lend itself to a meta-analysis. There are very few randomized controlled trials (RCTs) and the measures are quite different between the limited RCTs. However, we did not want to exclude articles from our review that were not RCTs as we believe they contribute meaningful information to our objective. In addition, this review provides insight on how to proceed both clinically and in future research.

All 8 RCTs used the comparators of in-person, usual care. Standard in-person usual care could vary from location to location and from country to country. No studies were found that compared different types of VC to determine the most effective method/type/intervention. The studies reviewed focused on patients from multiple populations with a differing chronic illnesses. While VC has been used in a wide range of health problems, reported studies using VC focus on one specific disease. VC has potential for use in patients with MCC and health disparities in multiple healthcare settings. Addressing the confounding co-morbid conditions experienced by many individuals living with chronic illness should be a priority. Future, prospective randomized controlled trials with adequate power that incorporate VC into Primary care and focus on MCC are warranted. Designing these trials will be complex and should be done using an organizing framework for developing complex interventions.

## Figures and Tables

**Table 1:** Literature review matrix: study aim, design, type of video conferencing (vc), sample, data collection characteristics, chronic illness, and results.

(Authors, Year) Location	Aim	Design	Type of Video Conferencing (VC)	Sample Size/ (Description)	Data Collection	Condition	Results
(Amarendran, George, Gersappe, Krishnaswamy, & Warren, 2011) United States	To assess the differences between using VC verses In-person assessment of movement using the Abnormal Involuntary Movement Scale.	Quasi-experimental Correlational/Case-control	Dedicated equipment with an ISDN connection.	N = 50 (male = 47) patients in the VA system with a history of antipsychotic medications for at least 10 years.	Abnormal Involuntary Movement Scale (AIMS).	Mental Health	There are no significant differences between VC and In-person assessment of involuntary movement using the AIMS assessment tool.
(Azad, Amos, Milne, & Power, 2012) Canada	To evaluate VC use in a follow up clinic for patients with memory disorder living in rural areas.	Descriptive feasibility	Web based VC is assumed because of reference to “Video Link” in the article.	N = 50 patients with mild to moderate memory disorder without functional changes.	Surveys developed by the study team.	Mental Health	Positive patient perceptions of VC. Measurements included: being understood by providers, having enough time, getting questions answered, and being the same as an in-person visit.
(Azar *et al.*, 2015) United States	To evaluate the use group VC to deliver a lifestyle intervention to virtual small groups and to compare the change in body weight and BMI from baseline to 3 months.	RCT	Web based group visits and weekly Bluetooth scale measurements.	64 total (Men ages 21 – 60 BMI between 30 – 40, no type 1 diabetes or serious medical condition or taking weight loss medication or participating in medically supervised weigh loss program) 32 Intervention 32 control.	Demographics via questionnaire Height, Weight, BP via automated cuff. Intervention: Weight via Bluetooth scale weekly, attendance at video visit, self-monitoring of body weight.	Obesity	Participants in the intervention group lost significantly more weight, 3.5% (95% CI 2.1%, 4.8%), than those randomized to the control group. Participants attended 9 of 12 sessions on average and weighed themselves at least once per week over the course of the intervention.
(Chua, Craig, Wootton, & Patterson, 2001) United Kingdom	To compare VC to In person new patient neurology referrals	RCT	Video conferencing via phone lines/SDN.	N = 168 (VC = 86, In-person = 82) newly referred by PCP to non-urgent Neurologist visits in two hospital centers in the UK.	Number of assessment, number of medications prescribed, and review of history, patient satisfaction, and diagnostic categories.	Neurology	VC was less efficient and not as well received by patients than In-person care.
(Dorsey *et al*., 2013) United States	To evaluate the feasibility, effectiveness, and economic benefits of VC care for persons with Parkinson disease in their home.	RCT	Web Based VC	N = 20 (VC = 9, control = 11) patients with Parkinson disease and Internet access at home.	Percentage of VC visits completed as scheduled, Parkinson Disease Questionnaire, time, and travel.	Neurology	VC offers similar clinical control and saved participants 100 miles of travel and 3 hours of time.
(Grady, 2002) United States	To compare the costs to patients, medical system, and organization of four methods of mental healthcare in military medical clinics.	Cost analysis	Dedicated equipment with an ISDN connection.	Not stated	Cost analysis	Mental Health	The least expensive method of mental healthcare delivery was tele-mental health care using video conferencing.
(Grady & Melcer, 2005) United States	Compare treatment and outcomes of mental health care via VC to in-person care.	Retrospective chart review	Video conferencing via phone lines/ISDN.	N = 81 (VC = 51 and in-person = 30) Adult patients in the VA system Seeking mental health care between April 1, 1999 to March 31, 2000.	The Global Assessment of Functioning scale, Laboratory studies, number of medications, compliance, mental status examination, recommendations to utilize resources, and general number and type of diagnosis, behavioral characteristics of the psychiatrists.	Mental Health	Global assessment of functioning and compliance was statistically significant for improved for the VC group as compared to in-person interactions. No significant differences in number of tests, self-help recommendations, assessments or numbers of medications were seen.
(Hailey, 2008) Canada	To review evidence related to clinical and administrative outcome of tele-mental health studies.	Review article	Specific VC type for each article Not discussed.	72 published papers “Conducted in a scientifically valid Manner” reporting clinical or administrative outcomes controlled studies VC was compared with a non-VC alternative and Non-controlled studies 20 or more subjects related to tele-mental health.	General mental health, depression, panic disorder, smoking, cognitive disability, pediatrics, OCD, schizophrenia, substance abuse, eating disorders, suicide prevention, PTSD.	Mental Health	The quality of VC studies was limited with most being preliminary. The two RCTs in the paper found no difference in quality of VC verses In-person and one non-random study found improved mental health outcomes for VC compared to In-person encounters.
(Khatri, Marziali, Tchernikov, & Shepherd, 2014) Canada	To compare the provision and outcomes of group cognitive behavioral therapy when delivered using VC as compared to in-person delivery.	Non-randomized Pre/post mixed methods quasi experimental (Allowed participants to choose VC or In-person).	Web based VC	N = 18 adults (8 = VC, 10 = In-person) with diagnosis of mood, anxiety disorder, and/or adjustment disorder with access to a computer, webcam, and internet.	Beck Depression Inventory Second Edition and Qualitative theme analysis	Mental Health	BDI-II scores and qualitative analysis of the themes were similar across the two delivery formats. VC is comparable to in-person group based cognitive therapy.
(Kitamura, Zurawel-Balaur a, & Wong, 2010) Canada	Use systematic review of the literature to evaluate the feasibility of assessing, monitoring, and managing oncology patients via video conferencing.	Systematic review	Specific VC type for each article Not discussed.	N = 19 published articles of 15 clinical oncology patient groups: one small RCT; 7 non-randomized with control groups, and 7 case studies. Total 709 VC study patients and 346 control patients.	Reported outcomes included patient satisfaction (no validated scales) and preference for VC consultation, costs, provider satisfaction and convenience. accessibility of care and clinical outcomes limited.	Oncology	VC is feasible, effective for assessing, monitoring, and managing oncology patients, and clinical outcomes were not compromised; time and cost were comparable or reduced. Limited power of inference with small samples and methodological weaknesses.
(Lewis, 2003) United States	To evaluate a web monitoring system intended to improve walking ability post-stroke.	Case-Study	Web based VC system with integrated performance indicators.	N = 2	Post-satisfaction questionnaire.	Rehabilitation	One participant evaluated the system favorable and one participant wanted an in person therapist.
(Lipman, Kenny, & Marziali, 2011) Canada	To evaluate the feasibility of providing web-based support and education for single mothers.	Pre/post mixed methods descriptive quasi experimental.	Web Based VC	N= 15 single mothers with health disparity having children ages 3 – 9.	Qualitative interviews, demographic, medications, CES-D, Rosenberg Self-Esteem Scale, Social Provisions Scale, and Parenting Stress Index-Short Form.	Mental Health	Positive perceptions of the VC intervention via qualitative evaluation and improvement of all quantitative outcome measures.
(Man, Soong, Tam, & Hui-Chan, 2005) Hong Kong, China	Comparing the effectiveness of online VC with interactive software, interactive software alone, in-person and control for problem-solving skill training groups for persons with Brain Injury.	Comparative effectiveness Pre/post quasi experimental	Web Based VC	N = 109 person with Acquired Brain Injury in Hong Kong.	Problem-solving skills, Activities of Daily living.	Neurology/Brain Injury	VC with therapist-administered group was effective in improving problem solving skills in persons with ABI.
(Marhefka *et al*., 2013) United States	To evaluate participant satisfaction, facilitators, experiences and technology of video group delivery of the Healthy Relationships intervention for women living with HIV.	Qualitative	Video education using a terminal at primary care clinics to connect to a remote education group.	N = 4	Demographics, qualitative discussion and open-ended questionnaire.	HIV	Video group participation was feasible and valued by participants. Efficacy was not evaluated.
(Martin-Khan *et al*., 2012) Australia	Evaluating the use of VC versus standard in-person care to establish a diagnosis of dementia.	RCT	VC with ISDN connection.	N = 205 (VC = 100 In-person = 105) patients aged 50 or older referred for cognitive assessment.	Mini-Mental State Examination (MMSE), Rowland Universal Dementia Assessment Scale (RUDAS), Clock Face Test (CFT), Letter Naming Verbal Fluency Test (FAS), Naming Animals Verbal Fluency, Geriatric Depression Scale-15 questions (GDS-15), Informant Questionnaire on Cognitive Decline in the Elderly (IQCODE), Neuropsychiatric Inventory Short form (NPI-Q), and Disability. Assessment for Dementia (DAD).	Mental Health	In general, there was agreement between the VC and in-person assessments with only 1% difference between the total scores for overall agreement.
(Marziali & Donahue, 2006) Canada	To compare the effectiveness of an internet based VC support intervention to a no treatment group in a sample of caregivers of older adults with neurodegenerative disease.	RCT	Web Based VC	N = 66 caregivers of older adults with Alzheimer’s, stroke related dementia, and Parkinson’s.	Health Status Questionnaire 12, abbreviated Medical Outcomes Study 36, Center for Epidemiologic Studies-Depression scale, self-report of depressive affect and behavior, instrumental ADLs, Revised Memory and Behavior Problems Checklist, and Multi-dimensional Scale of Perceived Social Support.	Neuro/Mental Health/Caregivers	Over half of the caregivers had never used computers but reported that the training was sufficient and 78% indicated the website was easy to use. 95% rated using the computers as a positive experience. At least one participant reported that VC was more helpful than in-person. The VC group improved on reported stress- and the control group worsened. There was significantly higher attrition in the control group.
(Marziali & Garcia, 2011) Canada	To compare dementia caregivers’ stress and health status when enrolled in one of two groups: internet chat support that included caregiver handbook and 6 videos on managing caregiving versus Internet based VC delivered by a clinician with access to caregiver handbook, support.	Non-randomized Comparative effectiveness pre/post design quasi experimental.	Web Based VC	N = 91 (Chat Group N = 40; Video Group N = 51).	Demographic information, Eysenck Personality Questionnaire-Revised, neuroticism, Revised Scale for Caregiver Self-efficacy, beliefs about caregiving, Perceived Social Support, Health Status Questionnaire, Center for Epidemiologic Studies Depression Scale, Functional Autonomy Measurement System, current outside service use, and intent to continue caregiving at home.	Mental Health/Caregivers	Both groups significantly improved in self-efficacy. Neither group changed in use of health and social services. When compared, the VC group had greater improvement in mental health and reported distress scores but the Chat group had lower distress reported for managing Instrumental Activities of Daily Living (IADLs).
(Norman, 2006) United Kingdom	To review the evidence related to the use of VC for mental health issues in the United Kingdom.	Review article	Specific VC type for each article Not discussed	72 Abstracts	Efficacy, cost-effectiveness, and satisfaction.	Mental Health	VC has been cost effective and reliable method for patients with mental health issues. Limitations still exist that need to be addressed including type of patients and confidentiality.
(Peel, Russell, & Gray, 2011) Australia	To evaluate an in-home VC system called eHAB for feasibility of home rehabilitation to older adults.	Feasibility	Web based VC system	N = 44	No actual participants were recruited and monitored using the VC system.	Rehabilitation	This VC system was not feasible in an older population with rehabilitation needs. Special needs of this population require an easy to use light and mobile system or in home support to operate the equipment. The unique needs including decreased vision, hearing, and decreased physical mobility need to be addressed.
(Somers *et al*., 2015) United States	Using VC on a tablet computer to deliver a brief Pain Coping Skills Training (PCST) for patients with persistent pain from cancer.	Pre/post design for feasibility and acceptability quasi experimental.	Web Based VC (Skype) on a tablet computer.	6 male and 19 female patients with cancer; mean age of 53.9 + 12.6 yrs.	Measures included pain, physical functioning and symptoms, psychological distress, self-efficacy for pain management and pain catastrophizing via pre-and post-intervention questionnaires. collected on a secure website via the mobile tablet.	Oncology	18 of the 25 participants completed all 4 sessions and 1 completed 3 sessions with post-intervention outcome data for the 19; video conferencing was feasible and acceptable. Pre-post interventions scores showed significantly decreased pain severity, physical symptoms, psychological distress, pain catastrophizing. Limited generalizability with small, non-randomized samples.
(Temple, Drummond, Valiquette, & Jozsvai, 2010) Canada	To compare assessment of persons with intellectual disability (ID) using VC to in person assessments.	Descriptive observational	Encrypted web based VC	N = 19 adults (23 – 63) with Intellectual disability.	Wechsler Abbreviated Scale of Intelligence and the Beery-Buktenica Developmental Test of Visual-Motor Integration-IV.	Mental Health	There are no statistically significant differences between assessment of ID between VC and in-person assessment.
(Vadheim *et al*., 2010) United States	To assess the feasibility of delivering a Diabetes Prevention Program group intervention through VC versus In-person.	Descriptive/Case-control	Not described	N = 19	Attendance, completion, weight, blood glucose, lipid values, current medication, self-monitoring, dietary intake.	Diabetes	All participants completed the In-person group and 88% completed the telehealth group. All participants mproved biophysical measurements and there was not statistical difference between the VC and In-person group.
(Wakefield, Buresh, Flanagan, & Kienzle, 2004) United States	To assess satisfaction and outcomes of VC for specialty care for residents of a long-term care center.	Descriptive	VC with ISDN connection provided by the Iowa Communications Network.	N = 76 patients living in a nursing home and needing a specialty medical consultation appointment.	Outcomes (Change in treatment yet remaining at the care facility, no change in treatment and remain at care facility, other), Satisfaction with VC.	Long-term care	There was a high level of satisfaction for both patients and providers. VC allowed most patients to remain in the long-term care facility instead of having to leave for specialty appointment.
(Weiner *et al*., 2003) United States	To assess patient and provider satisfaction with unscheduled VC for persons living in a Nursing home.	RCT-this article presents early findings from intervention group.	Modem Web Based VC	N = 187 patients living in a nursing home.	Patient characteristics, reason for VC, satisfaction.	Long-term care	Medical decision-making was easier via VC verses phone consultation. No patient reported that VC communication was different than usual care.
(Wong, Martin-Khan, Rowland, Varghese, & Gray, 2011) Australia	To validate the RUDAS dementia screening via video conferencing.	RCT	Video conferencing with simulated Limited bandwidth connection using a CODEC devices.	N = 42 Mean age was 74.8 years with a mean MMSE of 24.7, 8 tested positive for dementia.	Age, Mini-Mental State Examination (MMSE), RUDAS.	Neurology	There is no statistically significant difference in mean RUDAS scores for in-person or Video Conference administered assessments at both the total score, and individual domain levels. Hence the RUDAS can be reliability administered and scored via Video conference.
(Woodend *et al*., 2008) Canada	To evaluate the effect on healthcare resource use, morbidity, and quality of life, of a 3 month intervention that included video conferencing enhanced telemonitoring for patients with heart failure (HF) or angina.	RCT	3 months of weekly video conferencing with a nurse in addition to daily telephone transmission of weight, blood pressure and periodic electrocardiograms (EKG) and a 1-year end-of study assessment.	N = 249 (121 HF/28 angina) with 70% male participants; mean age of 66 ± 12 yrs.	Primary outcome: hospital readmissions and days in hospital. Secondary outcomes: morbidity assessed by weight, blood pressure, ECG; quality of life (SF36), functional status (The Minnesota Living with Heart Failure Questionnaire and the Seattle Angina Questionnaire).	Angina or HF	VC in combination with other home monitoring was easy to use and had high satisfaction; outcomes for patient recall data documented reduced number of hospital readmissions & days in hospital for patients with angina, and improved quality of life and FS for both groups: HF and angina. No significant differences in physician visits beween VC and usual care groups. The type of monitor is not reported nor the % of time that VC transmission problems resulted in telephone interviews only.
(Woolf et al., 2015) United Kingdom	Assess the feasibility for comparing remote therapies for word finding for persons with aphasia in four groups; two remote sites (University and Clinical), in-person therapy, and a attention control.	Feasibility	Web Based VC	N = 21 people with aphasia after left hemisphere stroke.	Feasibility and word retrieval via picture naming and conversation.	Neurology/Stroke	Participants gave good ratings for connectivity and for visual and sound quality. They problem-solved when needed by moving the equipment or redialing. Participants in the therapy groups reported independently practicing. Compliance and Participants in all groups improved but those who received remote therapy from the clinical site were most improved.

## List of Abbreviations

ABI: Acquired Brain Injury
AIMS: Abnormal Involuntary Movement Scale
BDI-II: Beck Depression Inventory Version 2
BMI: Body Mass Index
BP: Blood Pressure
CBT: Cognitive behavioral Therapy
CES-D: Center for Epidemiologic Studies Depression Scale
CFT: Clock Face Test
DAD: Disability Assessment for Dementia
EKG: Electrocardiograms
FS: Finger Stick
HF: Hear Failure
HIV: Human Immunodeficiency Virus
ID: Intellectual Disability
IQ: Intelligence Quotient
ISDN: Integrated Services Digital Network
IQCODE: Informant Questionnaire on Cognitive Decline in the Elderly
GSD-15: Geriatric Depression Scale-15 Questions
MCC: Multiple Chronic Conditions
MMSE: Mini-Mental State Examination
NPI-Q: Neuropsychiatric Inventory Short form
OCD: Obsessive Compulsive Disorder
PC: Primary Care provider
PCST: Pain Coping Skills Training
PTSD: Post Traumatic Stress Disorder
RCT: Randomized controlled Trial
RUDAS: Rowland Universal Dementia Assessment Scale
SF36: Short Form Health Survey 36 questions
VA: Veterans Administration
VC: Video Conferencing
VMI: Beery-Buktenice Developmental Test of Visual-Motor Integration-IV
WASI: Wechsler Abbreviated Scale of Intelligence
